# Winter Dietary Analysis Reveals the Foraging Differences of Wild Boar (*Sus scrofa*) in Different Regions of a Karst Mountainous Area

**DOI:** 10.3390/ani13040727

**Published:** 2023-02-17

**Authors:** Heqin Cao, Xiongwei Yang, Caichun Peng, Yeying Wang, Qunyi Guo, Haijun Su

**Affiliations:** 1 Forestry College of Guizhou University, Guiyang 550025, China; 2 Research Center for Bio-Diversity and Nature Conservation, Guizhou University, Guiyang 550025, China; 3 Guizhou Karst Environmental Ecosystems Observation and Research Station, Ministry of Education, Guiyang 550025, China; 4 Aha Lake National Wetland Park Management Office, Guiyang 550002, China; 5 College of Life Science, Guizhou Normal University, Guiyang 550001, China; 6 Administration of Wildlife and Forest Plants of Guizhou Province, Guiyang 550001, China

**Keywords:** wild boars, DNA metabarcoding, dietary composition characteristics, dietary differences, mountainous karst areas

## Abstract

**Simple Summary:**

Studying the dietary habits of animals helps us to understand the food composition requirements and nutrition strategy of these animals, as well as allows us to explore their resource utilization and relationship with their prey. Due to the difficulty in obtaining samples of the stomach contents of wild boars, there are few reports on the feeding habits of wild boars in karst areas. In this study, the stomach contents of 14 south China wild boars were analyzed by DNA metabarcoding. The results showed that there were 153 genera, 93 families, and 48 orders of plant food sources. *Cissus*, *Dioscorea*, *Quercus*, *Actinidia*, and *Houttuynia* were the core plants. The remaining foods were animal food sources, including invertebrates and vertebrates. It is noteworthy that wild boars in different regions also showed different foraging preferences in winter. This study revealed the foraging preference of wild boars under the special forest vegetation conditions in the mountainous area of southwest China, as well as the relationship between the food habits of wild boars and their habitats from the perspective of resource utilization, thus providing a key scientific basis for the prevention and control of wild boars, along with resource protection.

**Abstract:**

Wild boars (*Sus scrofa*) are extremely common in southern China, but little study has been conducted regarding reporting the dietary habits of wild boars using high-throughput sequencing technology, especially in karst areas, due to the difficulty in obtaining stomach contents of wild boars. In our study, the stomach contents of 14 wild boars in southern China were analyzed by DNA metabarcoding. The results showed that there were 153 genera, 93 families, and 48 orders of plant food sources for wild boars. The main plant food component were *Cissus, Dioscorea, Quercus, Actinidia*, and *Houttuynia*. The most numerous taxa of animal food sources were *Elaphodus, Amynthas, Chonaphe, Rattus,* and *Tanytarsus*. It is noteworthy that *Elaphodus cephalophus* were detected in most of the stomach samples, accounting for a large portion of animal food sources. The results showed that there were significant differences in the diets of wild boars in different regions; however, no significant differences were noted between male and female wild boars. Our study revealed the dietary preference of wild boars under the special forest conditions in the mountainous area of southwest China, as well as the relationship between the dietary habits of wild boars and their habitats from the perspective of resource utilization, thus providing a key scientific basis for the prevention and control of wild boars, along with resource protection.

## 1. Introduction

Wild boars (*Sus scrofa*), belonging to Vertebrates, Mammalia, Artiodactyla, Suidae and *Sus*, are widespread large mammals inhabiting the south of the Yangtze River in China [1]. They are widespread largely due to their ability to survive well in changing environments. With the rapid increase in the wild boar population in recent years, there are currently no good prevention and control strategies, leading to increased hazards. Numerous studies have been carried out on the ecology of wild boars, at home and abroad, involving population density, habitat selection, activity rhythm, home range, etc. [2,3,4]. However, there are relatively few reports on the dietary habits of wild boars in south China, which is a large and widely distributed area, and there is little understanding of their ecological roles and interactions in nature.

The analysis of dietary composition and nutritional characteristics can help to understand how wild boars use different habitats to determine their role in the food web. It is also a necessary condition for exploring the coexistence of species competition [5]. As we all known, the wild boar is an omnivore, with its main foods including oak fruits, roots, green leaves and crops, insects, earthworms, birds, small beasts, etc. The dietary habits of wild boars may also vary regionally and seasonally, largely depending on their food availability [6]. The food availability affects not only the diet of wild boars, but also population dynamics, habitat use, diffusion, reproduction, and interactions with other species [2,7].

Learning the traditional dietary habits involves direct observation and the microhistological analysis of feces. The traditional direct observation method is difficult due to the challenge of systematically tracking animals with high alertness, while the microhistological analysis of feces is less efficient in identifying food types with similar morphology, and it is difficult to identify animal-based foods with high digestibility [8]. With the continuous improvement of investigations of animal food habits and the corresponding research methods, high-throughput sequencing technology with high resolution and comprehensive coverage of information has been gradually applied to research on the food habits of a variety of animals [9]. We explored the relationship between the diet and habitat of wild boars in the mountainous areas of southwestern China using DNA metabarcoding, hoping to provide a reliable scientific basis for the prevention and control of wild boar hazards, as well as resource protection [10,11]. We aim to reveal (1) the dietary habits of wild boars in karst mountains in winter; (2) differences in dietary habits of wild boars in different regions of karst mountains in winter; (3) differences in dietary habits between male and female wild boars in karst mountainous areas in winter.

## 2. Materials and Methods

### 2.1. Study Area and Sample Collection

Guizhou province, located in the eastern Yunnan-Guizhou plateau in southwestern China, boasts the world’s most extensive, concentrated, and typical karst landscape, accounting for 61.9% of the province’s total land area. The Miaoling Mountains traverse the central part of the territory and are the watershed between the Yangtze River and the Pearl River. The northern portion of the Miaoling Mountains belongs to the Yangtze River Basin, and the southern portion belongs to the Pearl River Basin. The karst landforms in the northern Miaoling Mountains account for more than 60% of the area, with undulating and rugged terrain, barren soil, and weak water storage capacity. The southeast region of the Miaoling Mountains is a non-karst region, with superior soil and water conditions, which lays a good foundation for the richness and diversity of plants [12].

**Figure 1:**
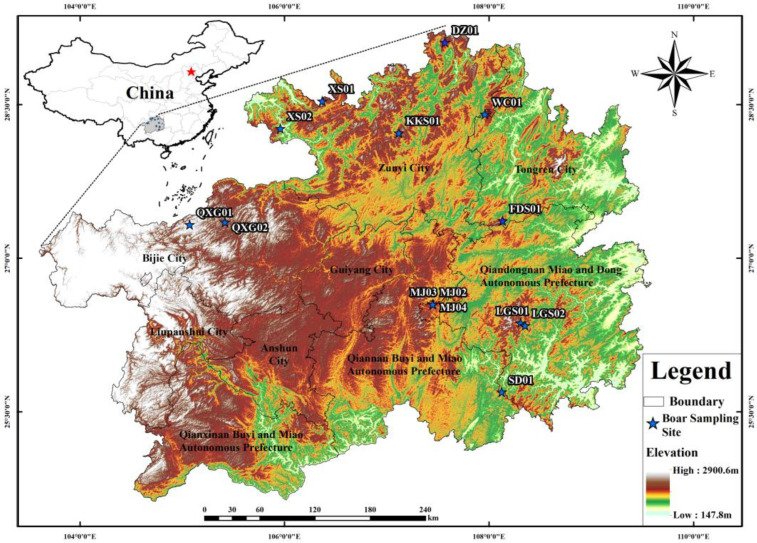
Sampling sites of 14 healthy wild boars.

In the winter of 2019–2020 (Dec-2019, Jan-2020, Feb-2020), 14 healthy wild boars (8♂, 6♀) with an average weight of 61.16 ± 8.95 kg were captured from the southwest karst mountainous region in China (Figure 1), and divided into the northern area (N) and the southern area (S), with the Miaoling Mountains as the boundary (Table S1). After the wild boars were killed, their stomachs were ligated at both ends, and 6–10 g of stomach contents were collected with a sterile scalpel or forceps, then immediately put into 15 mL sterile sampling tubes for labeling and immediately frozen in liquid nitrogen to ensure that the microbes were not polluted by the environment. Then, the samples were stored at −80 °C for the next sequencing experiment [11,13]. The hunting and autopsy sampling was approved by the Guizhou Forestry Bureau and the Animal Experiment Ethics Committee of Guizhou University.

### 2.2. DNA Extraction

The total genomic DNA was extracted using the OMEGA soil DNA kit (Omega Bio-Tek, Norcross, GA, USA), following the manufacturer’s instructions, and stored at −20 °C prior to further analysis. The quantity and quality of the extracted DNAs were measured using a NanoDrop NC2000 spectrophotometer (Thermo Fisher Scientific, Waltham, MA, USA) and agarose gel electrophoresis, respectively.

### 2.3. Amplicon Sequencing

Using the extracted DNA as a template, the chloroplast rbcL gene primers Z1aF/hp2R and mitochondrial cytochrome C oxidase subunit I (COI) gene primers COI intF/COI jgHCO2198 were amplified by PCR. The details of the primers are shown in Table 1.

The PCR components contained 5 μL of buffer (5×), 0.25 μL of Fast pfu DNA Polymerase (5 U/μL), 2 μL (2.5 mM) of dNTPs, 1 μL (10 uM) each of Forward and Reverse primer, 1 μL of DNA Template, and 14.75 μL of ddH2O. Thermal cycling consisted of initial denaturation at 98 °C for 5 min, followed by 25 cycles consisting of denaturation at 98 °C for 30 s, annealing at 53 °C for 30 s, and extension at 72 °C for 45 s, with a final extension of 5 min at 72 °C. PCR amplicons were purified with Vazyme VAHTSTM DNA Clean Beads (Vazyme, Nanjing, China) and quantified using the Quant-iT PicoGreen dsDNA Assay Kit (Invitrogen, Carlsbad, CA, USA).

### 2.4. High-Throughput Sequencing and Data Processing

Illumina’s TruSeq Nano DNA LT Library Prep Kit was used to prepare the sequencing library. First, sequence end repair on the PCR (polymerase chain reaction)-amplified product was performed, with a sequencing adapter with an A base added to the 3’end of the sequence and an index sequence added to the 5’end of the sequence. Then, magnetic bead screening was used to remove the self-linked fragments of the adaptor. Finally, the final fragment selection and purification of the library was carried out by 2% agarose gel electrophoresis [17]. The qualified library was diluted in a gradient, mixed according to the required sequencing volume, and the MiSeq sequencer was used for paired-end sequencing. The entire experimental process above was performed by Shanghai Personal Biotechnology Co., Ltd.

### 2.5. DNA Sequence Analysis

For sequencing results, the OBITools package (http://metabarcoding.org/obitools) was used for sequence splicing, tag identification, redundant sequence removal, denoising, sequence classification, and other steps to complete sequence sorting and analysis. The DNA sequences obtained by sequencing were compared and analyzed in the online databases of NCBI (https://blast.ncbi.nlm.nih.gov/ accessed on 3 January 2021) and BOLDsystem (http://www.boldsystems.org/ accessed on 3 January 2021) to ensure that the obtained sequences were included in the wild boars’ diet. The sequences obtained above were merged with 97% sequence similarity to generate characteristic sequence operational taxonomic units (OTUs) and abundance data tables. The characteristic sequences of OTUs were compared with the reference sequences in the database to obtain the corresponding taxonomic information for each OTU. In addition, combined with local species information, the sequences were further aligned manually to improve the reliability of the results. The principles of species identification and classification of sequences include: (1) When the consistency of comparison results is not less than 99%, the most matched sequence only corresponds to a single species, and the species is locally distributed, the sequence is considered to be from the species; (2) When the consistency of the comparison results is not less than 99%, and the best matching sequence corresponds to multiple species, the species without local distribution is excluded first. If there is still more than one species, the identification results are recorded as the smallest taxon covering these species; (3) When the consistency of comparison results is between 95% and 99%, the species with the highest consistency and local distribution are counted, and the identification results are denoted as the minimum taxon covering these species; (4) Sequences whose consistency is less than 95% are discarded; and (5) Sequences with a combined difference of less than 2% are merged [18].

### 2.6. Bioinformatics and Statistical Analysis

We used relative read abundance (RRA) to estimate the dietary composition of wild boars [19]. RRA is the percentage of the sequence number of a certain food category in the total food sequence of the sample, which reflects the relative biomass; the calculation formula is as follows:

$$RRA_{i}=\frac{1}{N}\overset{N}{\underset{j=1}{\sum }}\frac{S_{ij}}{\sum_{i=1}^{T}S_{ij}}\times 100\%$$

where *T* is the number of dietary categories, *N* is the total number of valid samples, and *S_i,j_* is the sequence number of the dietary category *i* in sample *j*. The sum of the RRAs of all food groups is 100%.

The alpha diversity (Chao1 index and Shannon index) was calculated using Mothur (v1.30) software to measure the species abundance and diversity of the sample, visualized by box plots [20]. The non-parametric Kruskal–Wallis rank-sum test of independent samples was used to analyze the significant differences in alpha diversity among different genders and regions. Independent-sample *t*-tests (for the normally distributed data) or Mann–Whitney U tests (for the non-normally distributed data) were used to compare the data between groups (males vs females, south vs north). Based on unweighted uniFrac distance, a similarity analysis is performed to test the significance of differences between the groups, and principal coordinate analysis charts were drawn. Statistical comparisons were analyzed by the Statistical Package for Social Science program (SPSS22.0, Chicago, IL, USA). *p* < 0.05 was considered statistically significant.

## 3. Results

### 3.1. Winter Diet Composition of Wild Boars

A total of 1,813,485 original sequences and 1,473,904 effective sequences were obtained from the 14 stomach content samples of wild boars by means of the amplicon chloroplast rbcL gene. The sample sequence range was 54,609–127,384, and the average effective sequence of each sample was 105,278. A total of 1,384,906 original sequences and 1,336,953 effective sequences were obtained by means of the amplicon mitochondria COI gene. The sample sequence range was 66,414–121,422, and the average effective sequence of each sample was 97,157.

#### 3.1.1. Plant Food Sources of Wild Boars

**Figure 2:**
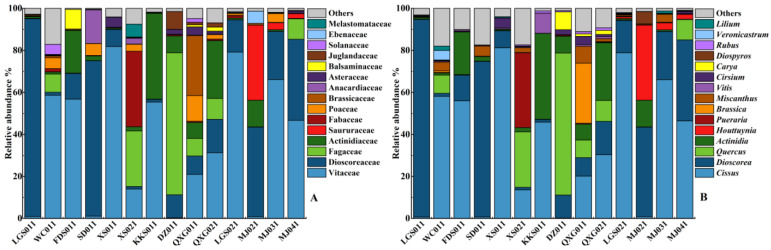
Relative plant food source abundance for wild boars at the (**A**) family level and (**B**) genus level.

A total of 45 orders, 94 families, and 153 genera of plants were identified, after optimized screening by removing their own sequences, as well as unidentifiable and small numbers of sequences, as plant food sources, of which only 64 were identified at the species level. At the family level (Figure 2), the top 10 plant food sources of wild boars at the family level were Vitaceae (36.74%), Dioscoreaceae (24.80%), Fagaceae (9.42%), Actinidiaceae (8.93%), Saururaceae (3.05%), Fabaceae (2.88%), Poaceae (2.54%), Brassicaceae (2.15%), Anacardiaceae (1.42%), and Asteraceae (1.01%). Among these, Vitisaceae, Dioscoreaceae, Fagaceae, Actinidiaceae, and Saururaceae are the most important winter food species for wild boars in the karst region, accounting for more than 82%. The abundance of ferns and bryophytes detected was extremely low.

At the genus level, *Cissus* and *Dioscorea*, accounted for more than half of the total detected. The most frequently detected plant food sources were plants from *Cissus*, accounting for 35.67%. The next-most frequently detected plant food sources were *Dioscorea* (24.80%). The other genera with a relative abundance greater than 1% include: *Quercus* (9.42%), Actinidia (8.93%), *Houttuynia* (3.04%), *Pueraria* (2.72%), and *Brassica* (2.13%), while *Miscanthus* (1.87%), *Vitis* (1.04%), *Cirsium* (0.99%), *Carya* (0.89%), *Diospyros* (0.48%), *Rubus* (0.37%), *Veronicastrum* (0.33%), and *Lilium* (0.28%) were consumed with relatively low frequency (Figure 2).

#### 3.1.2. Animal Food Sources for Wild Boars

**Figure 3:**
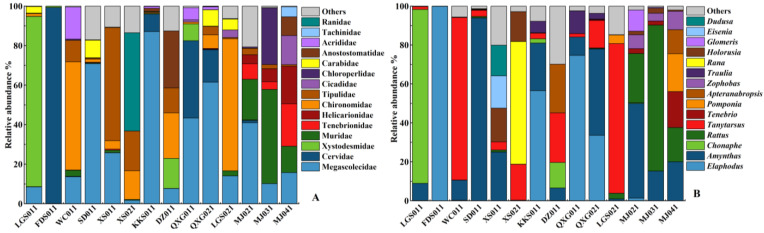
Relative animal food source abundance for wild boars at the (**A**) family level and (**B**) genus level.

A total of 4 phyla (Mollusca, Annelida, Arthropoda, and Chordata), 35 orders, 111 families, 117 genera, and only 60 species were identified as animal food sources (Figure 3), which were less diverse than plant food sources.

At the family level (Figure 3A), the invertebrates, such as Megascolecidae (35.15%), Xystodesmidae (10.23%), Tenebrionidae (3.76%), Helicarionidae (3.39%), and Chironomidae (2.68%), were the important winter animal food sources of wild boars. Mammals, such as Cervidae (23.65%) and Muridae (6.45%), are also an important component of the diet of wild boars, while Ranidae (0.72%) can only be detected in six samples, showing little consumption.

At the genus level (Figure 3B), the relative abundance of *Elaphodus, Amynthas, Chonaphe, Rattus*, and *Tanytarsus* were as high as 81%, making these the main animal food sources of wild boars. *Amynthas* and *Tanytarsus* were detected in all samples. *Elaphodus, Tenebrio, Pomponia, Apteranabropsis, Zophobas, Traulia, Rana,* and *Holorusia* were frequently detected as animal food sources, while a small proportion of *Glomeris, Eisenia*, and *Notodontidae* were detected as animal food sources, accounting for only 1.4%.

### 3.2. Winter Diet Composition of Male and Female Wild Boars

The main plant components of both male and female wild boars were *Vitaceae* and *Dioscoreaceae. Cissus, Dioscorea, Quercus,* and *Actinidia* made up more than 75% of the plant food for male and female boars. In general, female wild boars consumed slightly more animal food types than did males in winter, and Megascolecidae, Cervidae, and Helicarionidae were the main components of the diets of the female wild boars, accounting for 64%. The main food of male wild boars included Megascolecidae and Xystodesmidae, and it also contained more Cicadidae insects (Figure S1). 

**Figure 4:**
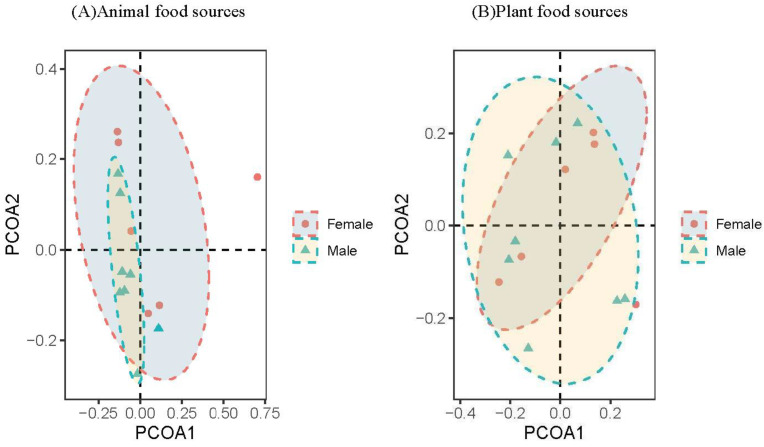
PCOA analysis for male and female wild boars.

**Figure 5:**
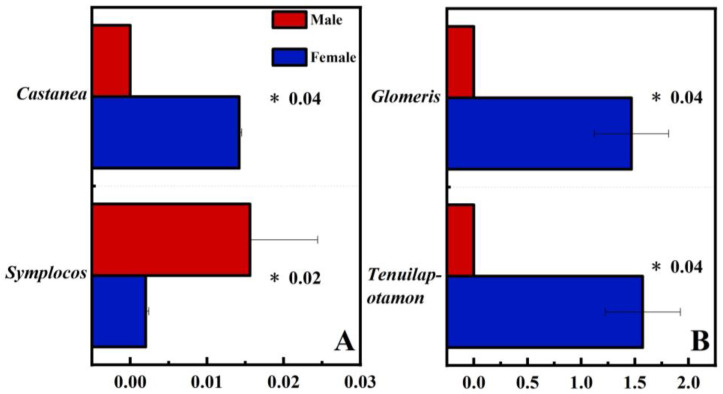
Comparison of relative abundances at the genus levels between male and female wild boars regarding (**A**) plant food sources and (**B**) animal food sources. * represents a significant difference *p* < 0.05.

The alpha diversity results showed that there was no significant difference between the diets of male and female wild boars (8♂, 6♀, Figure S2). PCOA analysis results showed that there were no significant differences between male and female wild boars for plant and animal food source composition (Figure 4). The results of differential analysis at the genus level showed that 2 genera showed significant differences between male and female wild boars regarding plant food sources composition, and the relative abundance of *Castanea* (*p* = 0.04) was significantly higher in female than in male wild boars. Moreover, regarding animal food sources composition, there were 2 genera that showed significant differences between male and female wild boars, including *Glomeris* (*p* = 0.04) and *Tenuilapotamon* (*p* = 0.04), which were significantly higher for female than for male wild boars (Figure 5).

### 3.3. Winter Diet Composition of Wild Boars in Different Regions

*Cissus, Vitis, Quercus, Actinidia*, and *Dioscorea* were the major plant foods of northern wild boars in winter, accounting for 74%, while *Dioscorea, Cissus,* and *Houttuynia* were the major plant components of the diets of northern boars, accounting for 87%. Cervidae, Megascolecidae, and Cicadidae dominated the animal food sources of the northern wild boars, while the dominant animal foods in the southern boars were the Xystodesmidae, Helicarionidae, and Megascolecidae (Figure S3).

**Figure 6:**
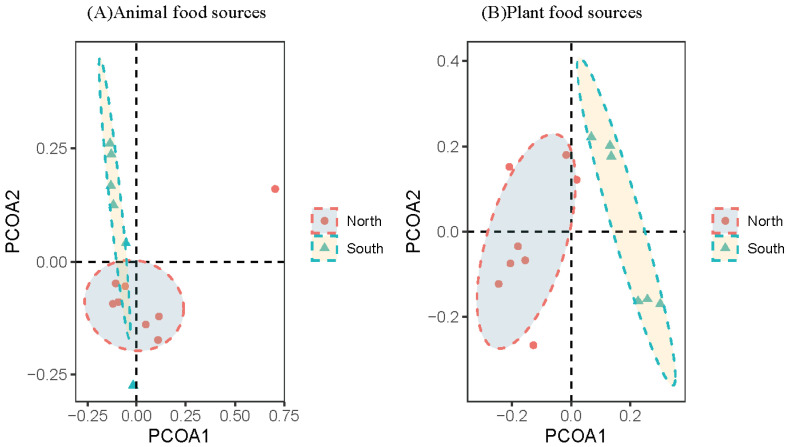
PCOA analysis of wild boars in different regions.

**Figure 7:**
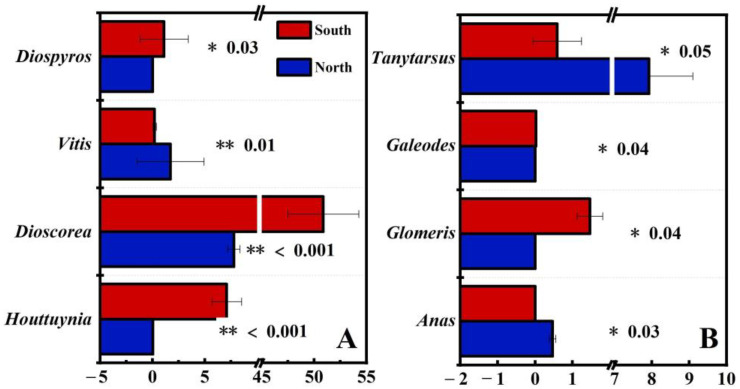
Comparison of relative abundances at the genus level between northern and southern wild boars regarding (**A**) plant food sources and (**B**) animal food sources. * represents a significant difference *p* < 0.05 and ** represents a significant difference *p* < 0.01.

There were no significant differences in the Chao1 index or the Shannon index in different regions (*p* > 0.05, Figure S4). PCOA analysis results showed that there were significant differences in different region regarding wild boars for plant and animal food sources composition (Figure 6). The results of differential analysis at the genus level showed that there were 4 genera that exhibited significant differences between the northern and southern wild boars in regards to plant food source composition, and the relative abundance of 3 genera, including *Diospyros* (*p* = 0.03), *Dioscorea* (*p* < 0.001), and *Houttuynia* (*p* < 0.001), was significantly higher for the southern than for the northern wild boars. Besides, there were 4 genera that showed significant differences between the northern and southern wild boars in regards to animal food source composition, and the relative abundance of 2 genera, including *Glomeris* (*p* = 0.04) and *Galeodes* (*p* = 0.04), was significantly higher for the southern as opposed to the northern wild boars (Figure 7).

## 4. Discussion

### 4.1. Composition and Structure of the Winter Diet of Wild Boars

Wild boars, omnivorous animals, are one of the most widely distributed mammals on earth. They have highly malleable food habits and opportunistic foraging characteristics. Due to their comprehensive adaptability to various kinds of foods, they can settle and reproduce in different habitat conditions [21,22,23]. Wild boars usually play different roles in the natural environment, such as predators, competitors, pollen/seed spreaders, and agricultural pests, affecting sympatric species by predation, destruction of nests, and habitat destruction, thereby changing the soil structure through their digging and foraging behavior. This leads to habitat degradation and the spread of diseases, which is closely related to the regional flora and fauna, as well as biodiversity [23,24,25]. Therefore, the study of dietary composition and nutritional characteristics helps in the understanding of how wild boars utilize different habitats, thus determining their role in the food web, and is also a necessary condition for discussing the competitive coexistence of species [2].

Our study revealed that wild boars in karst areas of China have a rich and diverse diet, including plants, animals, and unusual algae and fungi; these results are similar to those of Schley [6] and Ballari [24].

### 4.2. Plant Food Sources of Wild Boars

Our study revealed that wild boars appeared to be more dependent on plants than animals for food. Wang et al. found that in winter, wild boars in the Xiaoxing’an Mountains prefer plants such as *Equisetum hyemalein*, accounting for 87.61% of their diets [26]. This is related to the higher availability of plant food sources than animal food sources, resulting in greater dependence on plant food sources. *Cissus*, which is widely distributed in the area, is a major food source for wild boars, accounting for 35.67% of their diets. Moreover, its vine, root, and fruit are edible, and it also possesses medicinal value. Importantly, its fruiting berry lasts from November to May, thus *Cissus* become the most popular winter edible plants for wild boars, which is consistent with Li’s research [27].

*Quercus* and *Castanea* have always been important dietary components for wild boars everywhere. This kind of plant is rich in protein and sugar nutrition and is easy to digest; it is also a favorite food for many wild animals due to its nutritional value [7,23,28,29,30]. Tubers and kiwifruit are preferred foods of wild boars in winter, whether in this study area or in other research. Tubers contain the most abundant carbohydrate starch; total starch makes up to 85% of the total dry weight of tubers. Kiwifruit is fresh, tender, and juicy, with good flavor and high vitamin C content, making it generally favored by wild animals [7,30,31,32]. 

Incidents of crop damage have been commonly experienced in Europe and China [25,33]. In our study, the percentage of crop loss was low, for several reasons. It may be related to the sampling time and the low nutritional value and indigestion of the crops, or this result could also be caused by a boars’ highly seasonal diet [33,34].

### 4.3. Animal Food Sources of Wild Boars

As for animal food components, animal species that have been identified include invertebrates (snails, earthworms, insects, millipedes, spiders, crabs, centipedes, etc.) and vertebrates (small mammals, birds, amphibians, and fish). With their keen sense of smell, boars like to dig to locate underground animals for food. The animals in the soil are both rich in protein and highly available, becoming the main animal food sources for wild boars [7,24]. Several authors have emphasized the possibility that animal materials are underestimated in diets because of their high digestibility in scat analysis [35,36]. 

Ground-dwelling birds can also easily become food for wild boars because wild boars like to eat the eggs and young birds in nests on the ground. In our study, two ground-dwelling birds, *Anas platyrhynchos* and *Garrulax pectoralis*, were also detected in the stomach, with results similar to those of Ballari and Barrios [27]. Although a certain abundance of rodents was detected in multiple samples, most of them are nocturnal, alert, and difficult to catch, so they are unlikely to be common prey of wild boars. This result may be caused by the wild boars eating the carcasses of rodents or animals that eat rodents.

Interestingly, more than 70% of the samples detected *Elaphodus cephalophus* in the stomachs of wild boars. This may be the first evidence that wild boars may prey on *Elaphodus cephalophus* in China. There may be two potential explanations for these results. One is that in the southwestern karst area, there are only small and medium-sized carnivores, such as *Prionailurus bengalensis, Pagumalarvata,* and *Viverrazibetha*, etc., and the area is lacking in large top-predator species [36,37,38,39]. Wild boars may replace large carnivores as the top predators in karst areas, regulating the structure and function of ecosystems. Another explanation could relate to the carrion scavenging behaviors of wild boars. Some researchers have found that wild boars eat the carrion of European roe deer (*Capreous capreous*) and badgers (*Meles meles*), which are one of the most important scavengers in the forest ecosystem [40,41]. We also found a small number of frogs (*Rana hanluica, Xenophrys omeimontis,* and *Polypedates braueri*), fish, and crabs. However, in most studies, it has been found that amphibians and fish make up a small proportion of the diet of wild boars. In France, fish consumption has also been observed in dry rivers, where dying fish can easily become the prey of boars [34].

### 4.4. Food Composition and Structure of Male and Female Wild Boars

There was no significant difference in the alpha diversity and PCOA analysis, but there were 4 genera that showed significant differences between male and female wild boars. The proportion of *Actinidia, Cissus,* and *Quercus* are similar between male and female wild boars. Female wild boars liked to feed on root plants (*Dioscorea* and *Pueraria*) in winter, which is consistent with the results of previous studies. Wild boars in the natural environment of South Korea will also feed on root plants in winter until the coming of spring [41]. Wilcox et al. [42] also found that female wild boars in California, USA, prey on small mammals more frequently than do males. The energy requirements of female wild boars during pregnancy and lactation may drive them to obtain more energy by choosing more root plants and animal food sources, making up for the lack of carbohydrates and protein in the diet. Faced with the physiological cost of reproduction, female animals will actively seek a higher predation rate to balance their nutritional requirements [1,7,41]. In addition, females prefer to eat fewer crops than males, which may be due to the relatively low nutritional quality of grains and their lack of essential amino acids. The low-quality protein extracted from grains may not be sufficient to meet the nutritional needs of females during reproduction and lactation. It is also possible that males have more advantages in their physiological conditions and are more likely to obtain human-sourced food than females [33,42].

### 4.5. Food Composition and Dietary Structure of Wild Boars in Different Regions

There were no significant differences in alpha diversity, but PCOA analysis showed significant differences between the diets of wild boars in different regions. These results indicated that the foraging differences between wild boar in karst and non-karst areas can be distinguished by dietary composition. The reason for this difference is related to the vegetation types in the different regions. In addition to the high proportion of the *Cissus*, the proportion of plants of *Quercus, Actinidia*, and *Dioscorea* is relatively even among the northern wild boars, and the animal food sources came mainly from Cervidae and Megascolecidae, while in the southern region, Dioscorea, Cissus, and *Xystodesmidae* were dominant. This is consistent with Sun’s results, which revealed that the dietary composition of the wild boars also changes according to geographical changes because the more diverse the topography, vegetation structure, and complexity, the higher the diversity of plant and animal communities, and the higher the availability of food [43]. A total of 48 orders, 93 families, and 153 genera were identified in wild boars. The abundance and diversity of the identified plant species of wild boars were higher in southern China than those in the Ziwuling Mountains of Gansu, Korea, and Argentina [7,44,45]. This may be related to the different types and distribution areas of wild boars. Some researchers compared the foraging habits of wild boars in natural and urban environments and found that wild boars in natural environments consume more natural ingredients, while wild boars around cities consume more crops and human food waste [41]. It is precisely because of the strong adaptability of wild boars to different habitat conditions that the population can rapidly expand in a short time, causing crop damage and human–beast conflicts in various regions [25].

The availability of species-specific DNA barcoding depends on the quality of existing databases [9]. In the study, some species were identified only as families or genera because no species was identified from the sequences matched in the database. If a database could be built based on local species, the results would be more accurate.

Although DNA metabarcoding does not depend on the experience and subjective judgment of researchers, it greatly improves the accuracy and reliability of food species identification. The design of primers is very important, and it affects the accuracy of results; improper PCR operation will lead to false positives [46].

## 5. Conclusions

This study proved that the food sources of wild boars in mountainous karst areas of southwest China are complex and diverse, including plant food sources, animal food sources, and unusual algae and fungi. The types of winter foods consumed by male and female wild boars are generally similar. However, there were significant foraging differences between wild boars in karst and non-karst areas. Moreover, our results revealed that wild boars may have preyed on *Elaphodus cephalophus* and have the potential to be top predators in the karst mountains. In summary, wild boars also show strong adaptability in karst areas. This study provides some insight into the prevention and control of the wild boar population, contributing to the knowledge base from the perspective of wild boar feeding habits.

## Figures and Tables

**Table 1:** Sequences of primers used in this study.

Primer	Primer Sequence	Product Length	Source of Primer
ZlaF hp2R	5′-ATGTCACCACCAACAGAGACTAAAGC-3′ 5′-CGTCCTTTGTAACGATCAAG-3′	230 bp 230 bp	[14]
COIintF COIjgHCO2198	5′-GGWACWGGWTGAACWGTWTAYCCYCC-3′ 5′-TANACYTCNGGRTGNCCRAARAAYCA-3′	320 bp 320 bp	[15] [16]

## Data Availability

NCBI SRA: PRJNA836165.

## Supplementary Materials

The following supporting information can be downloaded at: https://www.mdpi.com/article/10.3390/ani13040727/s1, Table S1: Collection information regarding wild boar samples; Figure S1: Relative diet composition abundance of male and female wild boars at the genus level: (A) plant food sources, (B) animal food sources; Figure S2: The alpha diversity index of food habits of male and female wild boars: (A,B) plant food sources, (C,D) animal food sources; Figure S3: Relative diet composition abundance of northern and southern wild boars at the genus level: (A) plant food sources, (B) animal food sources; Figure S4: Comparison of alpha diversity of the dietary habits of wild boars in different regions: (A) plant food sources, (B) animal food sources.
