# Supplementary material for: Winter Dietary Analysis Reveals the Foraging Differences of Wild Boar (Sus scrofa) in Different Regions of a Karst Mountainous Area
Source: Animals (Basel). 2023 Feb 17;13(4):727. doi: 10.3390/ani13040727 (PMC9952271; doi:10.3390/ani13040727)
Supplement: Supplementary file 1 [file animals-13-00727-s001.zip › animals-2192693-supplementary.pdf]

Table S1: Collection information of wild boar samples

Number	Label	Sampling site	Coordinates	Date	Gender	Area
1	FDS011	Fodingshan National Nature Reserve	108°08'E; 27°21'N	2019.11	Female	N
2	WC011	Wuchuan County	107°57'E; 28°23'N	2019.11	Male	N
3	XS011	Xishui National Nature Reserve	106°22'E; 28°31'N	2019.12	Male	N
4	KKS011	Kuankuoshui National Nature Reserve	107°07'E; 28°13'N	2019.12	Male	N
5	XS021	Xishui County	105°57'E; 28°15'N	2019.12	Female	N
6	DZ011	Daozhen County	107°34'E; 29°06'N	2019.12	Male	N
7	QXG011	Bijie City	105°04'E; 27°19'N	2019.12	Male	N
8	QXG021	Bijie City	105°25'E; 27°20'N	2019.12	Female	N
9	LGS011	Leigongshan National Nature Reserve	108°18'E; 26°21'N	2019.11	Female	S
10	SD011	Sandu County	108°07'E; 25°41'N	2019.12	Male	S
11	LGS021	Leigongshan National Nature Reserve	108°21'E; 26°20'N	2019.12	Male	S
12	MJ021	Majiang County	107°27' E; 26°32' N	2020.02	Male	S
13	MJ031	Majiang County	107°27' E; 26°32' N	2020.02	Female	S
14	MJ041	Majiang County	107°27' E; 26°32' N	2020.02	Female	S

Note: N presents the North of Miaoling Mountain (Yangtze River System), S presents the South of Miaoling Mountain (Pearl River System)

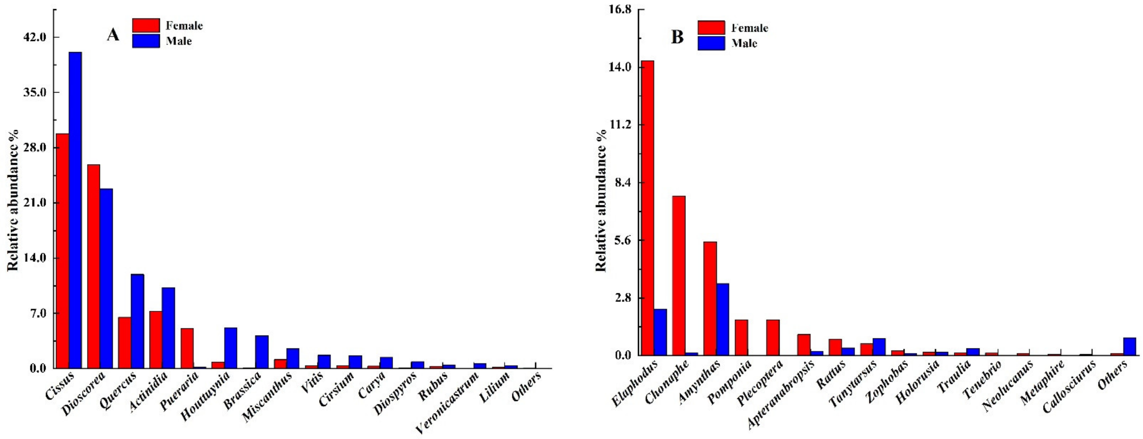

Figure S1 Relative diet composition abundance of male and female wild boars at the genus level (A) plant food sources (B) animal food sources

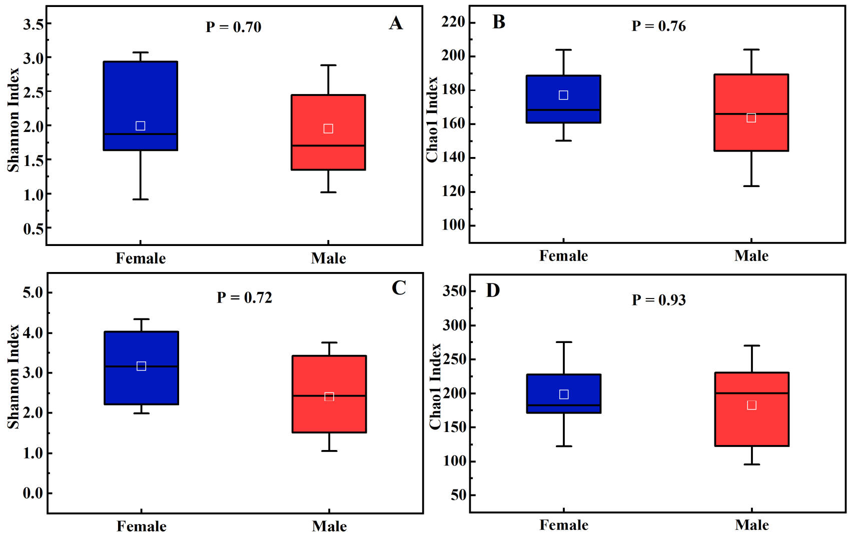

Figure S2 The alpha diversity index of food habits of male and female wild boars (A, B) Plant food sources (C, D) Animal food sources

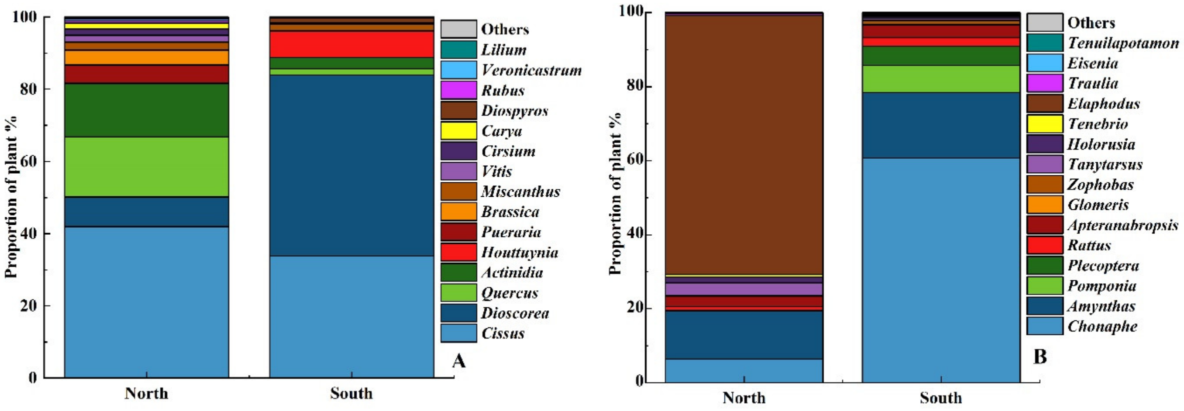

Figure S3 Relative diet composition abundance of northern and southern wild boars at genus level (A) Plant food sources (B) Animal food sources

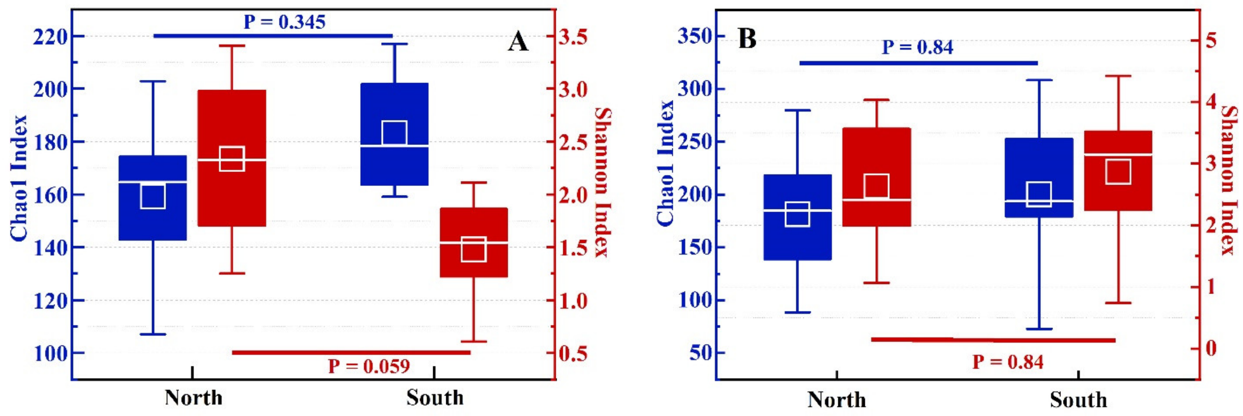

Figure S4 Comparison of alpha diversity of wild boars diet habits in different regions (A) Plant food sources (B) Animal food sources
